# Symptoms of anal incontinence and quality of life: a psychometric study of the Norwegian version of the ICIQ-B amongst hospital outpatients

**DOI:** 10.1186/s13690-022-01004-z

**Published:** 2022-12-09

**Authors:** Susan Saga, Anne Guttormsen Vinsnes, Christine Norton, Gørill Haugan

**Affiliations:** 1 Department of Public Health and Nursing, Faculty of Medicine and Health Sciences NTNU, Norwegian University of Science and Technology, Trondheim, Norway; 2 Florence Nightingale Faculty of Nursing, Midwifery and Palliative Care, King’s College London, London, UK; 3 Faculty of Nursing and Health Science, North University, Levanger, Norway

**Keywords:** Anal incontinence, Accidental bowel leakage, Faecal incontinence, Functional bowel disorders, Psychometric evaluation, Quality of life, Questionnaire, ICIQ-B, Bristol stool chart

## Abstract

**Background:**

The International Consultation on Incontinence Questionnaire-Bowel (ICIQ-B), a self-report, condition-specific questionnaire designed to assess symptoms of anal incontinence (AI), measures AI’s impact on quality of life (QoL) along with perceived bowel patterns and bowel control amongst individuals with AI. In our study, we aimed to translate the ICIQ-B to Norwegian and investigate the Norwegian version’s psychometric properties.

**Methods:**

To establish a relevant, comprehensive, and understandable Norwegian ICIQ-B, cognitive interviews were conducted with 10 patients with AI, and six clinical experts reviewed the translated scale. The Norwegian ICIQ-B’s structural validity, scale reliability, and content validity were tested amongst patients with AI attending hospital outpatient clinics in three regions of Norway (*N* = 208).

**Results:**

Assessing the Norwegian ICIQ-B’s content validity revealed that the questionnaire was relevant, comprehensive, and understandable. Missing data were infrequent (3.3%), and no floor or ceiling effects emerged. Three-factor and two-factor solution models, both with advantages and disadvantages, were found. The three-factor model offered the most parsimonious solution by covering most of the original scale, albeit with an unacceptably low reliability (α = .37) for the construct of bowel pattern. The two-factor model showed good reliability in terms of internal consistency for the constructs of bowel control (α = .80) and impact on QoL (α = .85) but was less parsimonious due to dismissing seven of the original 17 items and excluding the bowel pattern construct. Test–retest reliability demonstrates good stability for the Norwegian version, with an intra-class correlation coefficient of .90–.95 and weighted kappa of .39–.87 for single items.

**Conclusions:**

Although the Norwegian version of ICIQ-B demonstrates good stability and content validity, the original constructs of bowel pattern and bowel control had to be adapted, whereas the construct of impact on QoL remained unchanged. Further psychometric testing of the Norwegian ICIQ-B’s factor structure is therefore recommended.

**Supplementary Information:**

The online version contains supplementary material available at 10.1186/s13690-022-01004-z.

## Introduction

Anal incontinence (AI) is a debilitating condition that impacts an individual’s self-esteem and quality of life (QoL) and may cause significant secondary morbidity, disability, and economic burden [1]. In contrast to *faecal incontinence* (FI), defined as “the involuntary loss of liquid or solid stool that is a social or hygienic problem,” AI entails the involuntary loss of not only stool but also flatus from the rectum due to the inability to control bowel movements [2]. Thus, AI ranges from the occasional leakage of stool while passing gas to a complete loss of bowel control [3]. Because AI encompasses the loss of flatus and stool, its estimated pooled prevalence rate amongst home-dwelling adults is 15–17%, whereas FI’s is only 5.9% [4]. A population-based cross-sectional study among Norwegian women aged 30 and older found that 19.1% of the women reported AI, while 3.0% reported FI [5]. No studies have reported AI or FI prevalence among Norwegian home-dwelling men. However, because many patients avoid reporting FI, its prevalence may be underestimated [6, 4]. The highest prevalence is found among older people residing in care homes with an estimated FI prevalence of 42.8% [7].

The aetiology of AI is complex and multifactorial. Continence depends on the interaction between the anal sphincter complex, stool consistency, rectal reservoir function and neurological function. Disease processes or structural defects that alter any of those components can lead to FI [8]. Diarrhoea and altered bowel habits, inflammatory bowel disease, diet intolerance and constipation with paradoxical diarrhoea represent the most frequent independent risk factors for AI [9]. The most common structural causes, however, result from obstetrical injury [10], anorectal surgeries [11] and rectal prolapse [12, 13]. Depending on the presenting circumstances, FI is commonly classified as passive incontinence (i.e. involuntary discharge without any awareness), urge incontinence (i.e. discharge despite active attempts to retain it), and faecal seepage (i.e. leakage of stool with grossly normal continence and evacuation) [14, p. 1585].

Due to AI’s complex aetiology, treatment needs to be tailored to the individual’s circumstances [15]. Although several scoring systems are commonly used to assess AI, no investigative tools specifically link symptoms of AI to QoL [8]. For clinicians as well as researchers, validated questionnaires and scales play an integral role in identifying symptoms of a disease, assessing patients’ QoL, and objectively characterising any phenomenon detected [16]. Amongst such instruments, the International Consultation on Incontinence Questionnaire-Bowel (ICIQ-B) is a self-report, condition-specific questionnaire designed to assess symptoms of AI and its impact on QoL [17, 18]. As part of the International Consultation on Incontinence’s suite of validated questionnaires on incontinence [19], the ICIQ-B includes 21 main items, 17 of which address three scored factors: Bowel Pattern, Bowel Control, and Impact on QoL. In addition, to evaluate important issues from the perspectives of clinicians and patients, the ICIQ-B includes four unscored items: one representing the Bristol Stool Chart of stool consistency [20] and three others respectively concerning strain, worry and the restriction of sexual activities due to AI. Tailored for use by clinicians in both primary and secondary healthcare, the ICIQ-B is designed to screen for AI, obtain a brief yet comprehensive summary of the level, impact, and perceived cause of symptoms of AI and to facilitate better patient–clinician discussions [17, 18]. The ICIQ-B is intended for both clinical assessment and research. The 21 items are therefore divided in two parts; an A-question representing the main issue, accompanied by a B-question “*how much does this bother you*?” which is particularly important in a clinical perspective. The A-questions are measured on a 5- or 6-point Likert scale, while the B-questions are measured on a scale from (0 not at all) -10 (a great deal). One item, item 3, has a third question, since the main question regarding frequency of opening one’s bowels is further divided into a) usual and b) at worst and c) how much does this bother you? (Additional file 1).

Validated patient-reported outcome measures not only help patients and clinicians to make better decisions but also enable comparisons of providers’ performance to stimulate improvements in services. They are also well-suited for cross-national comparisons of research [21, 22]. To date, the ICIQ-B, originally developed in British English [17, 18], has been translated and validated in Spanish (i.e. in Chile), albeit only regarding content validity based on cognitive interviews [23]. Although an American English online version of the ICIQ-B has been psychometrically evaluated against an American English paper version [24], the extent of testing was limited. Even so, both cited studies involved assessing the test–retest reliability, which proved to be good in both cases [23, 24]. Moreover, the psychometric evaluation conducted in the United States demonstrated the ICIQ-B’s convergent validity and reasonable response to change at follow-up 3 months after the non-surgical treatment of FI, as well as its good internal consistency for the constructs of impact on QoL and bowel control. Meanwhile, having tested the American English version of the ICIQ-B, Markland et al. [24] demonstrated its fair internal consistency for the construct of bowel pattern. However, neither the Spanish nor the American English translation of the ICIQ-B has been assessed for structural validity. Beyond that, a review of QoL measures in relation to FI has shown that the original British English version of the ICIQ-B lacks sufficient structural validity [25]. Thus, because the ICIQ-B’s factor structure seems to be unclear, we evaluated the structural validity, reliability, and content validity of a Norwegian version of the scale.

### Aims

In our study, we aimed to translate the ICIQ-B to Norwegian and assess the translated scale’s psychometric properties amongst outpatients with AI. The research question was threefold:

1. How well does the original ICIQ-B’s three-factor measurement model fit with the observed data?
2. Does the ICIQ-B demonstrate good reliability in terms of internal consistency and test-retest stability?
3. Does the ICIQ-B demonstrate good content validity in the Norwegian population?

The research question was addressed in accordance with the COnsensus-based Standards for the selection of health Measurement Instruments (COSMIN guidelines) [26, 27], which address evidence related to structural validity, reliability, and content validity, all as central, interrelated properties of a given measurement model. Whereas *structural validity* (i.e. dimensionality) concerns the homogeneity of items [28]—that is, whether items match their respective constructs—*reliability* encompasses a scale’s inconsistency and lack of error [28]. By further contrast, *content validity* explores whether the theoretical content of constructs is adequately represented by questionnaire items in terms of relevance and comprehensiveness [29].

## Methods

### Translation and cultural adaptation

**Fig. 1:**
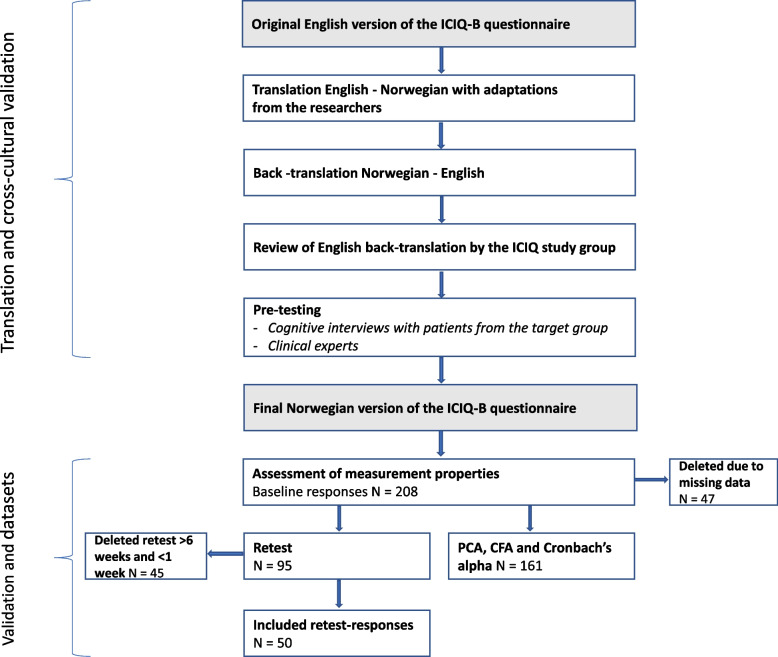
Flow chart of the ICIQ-B translation and validation in Norwegian

First, the ICIQ-B was translated from British English to Norwegian by a bilingual Norwegian–English translator, followed by a back-translation into English conducted by another bilingual Norwegian–English translator [19]. Second, the back-translation was evaluated by the International Consultation on Incontinence Questionnaire group [30]—that is, the British English instrument’s developers—who provided useful comments regarding possible ambiguities and other flaws that guided minor adjustments to the Norwegian ICIQ-B. Third, the Norwegian version was pilot-tested for comprehensiveness, readability, and equivalence [29] in cognitive interviews with 10 patients with AI living in Norway. Fourth, comments were gathered from six Norwegian bi- or monolingual multidisciplinary clinical experts to further assess comprehensiveness, readability, and equivalence. As minor discrepancies were identified and amended between each step, a comprehensible Norwegian version of the ICIQ-B gradually emerged (see Fig. 1).

### Participants and sampling procedure

In our study, three samples were recruited. The first was a sample of 10 patients, both men and women, recruited from the outpatient gastrointestinal surgery clinic of St. Olav’s University Hospital in Trondheim to participate in cognitive interviews. Patients with AI were invited to participate in the interview study by a nurse contact who provided them with information about the study, after which patients could contact the researcher directly. Written consent was obtained from the patients before their interviews commenced. Second, a sample of six clinical experts in AI (i.e. colorectal surgeons, stoma nurses and physiotherapists) from the three participating hospitals (i.e. St. Olav’s University Hospital in Trondheim, University Hospital Northern Norway in Tromsø and Akershus University Hospital in Oslo) were recruited to evaluate the Norwegian ICIQ-B’s comprehensiveness, relevance, and wording. The clinical experts were sent the Norwegian and original British English versions of the questionnaire via email, and the research team received their feedback either by email or orally during in-person meetings, depending on each expert’s preference. The cognitive interviews with patients and the evaluation by clinical experts were both part of pilot-testing the translated Norwegian version of the questionnaire and served to establish the foundation for the content validity and cultural equivalence between the Norwegian and British English versions of the ICIQ-B.

Third, to test the psychometric properties of the Norwegian ICIQ-B, patients referred from their general practitioners to outpatient clinics in the three mentioned university hospitals due to AI were recruited to complete a paper-based questionnaire. The three hospitals represent three regions of Norway from south to north. To be included, new patients had to be attending the outpatient clinic due to AI, had to have never received treatment for AI and had to be able to provide their written consent to participate in the study and to complete the questionnaire independently. Patients who participated in the cognitive interviews were not enrolled in that subsequent part of the study. A patient sample 10 times the number of items was needed to be able to perform a factor analysis of the Norwegian ICIQ-B [31]. Because the original questionnaire consists of 21 items, four of which are unscored and were excluded from our analysis, and because the remaining 17 items implied a sample size of approximately 170 patients, we aimed to include at least 200 questionnaire respondents.

Eligible patients were invited to participate by using the hospitals’ routines to summon patients. Along with an invitation to a medical consultation at the hospital, eligible patients received an information sheet about the study together with the questionnaire and a return envelope. Patients who attended the consultation subsequently received another invitation to participate in the study, which both reminded patients who had not yet responded to the questionnaire and served as a retest for those who had already returned their responses. Patients were recruited beginning in 2011 until 200 had been enrolled (i.e. in 2013).

### Analysis

Descriptive statistics and exploratory factor analysis (EFA) were performed with IBM’s SPSS version 28.0.1.0, while confirmatory factor analysis (CFA) was performed using Stata version 17.0 [32].

Structural validity was assessed of the main A-questions using CFA and EFA (Principal Axis Factoring). In our study, the model fit was assessed by χ^2^ statistics and two conventional fit indices—the root-mean-square error of approximation (RMSEA) and the standardised root mean square residual (SRMS)—with values less than .05 indicating a good fit and values from .05 to .10 indicating an acceptable fit [33, 34]. Furthermore, the comparative fit index (CFI) and the Tucker–Lewis index (TLI) with acceptable fit set at .95 and good fit at .97 were used [33–36]. Because skewness and kurtosis were significant, the Satorra–Bentler-corrected χ^2^ was applied as recommended when analysing non-normal continuous endogenous variables [37]. EFA was performed with oblim rotation, and observations with one or more missing values across the 17 variables included any of the three constructs were deleted. No replacements were made for missing data.

Next, content validity was assessed in three ways. First, cognitive interviews with patients in the target population and reviews of the scale by clinical experts were analysed on a question-by-question basis and any comments entered directly under each item on the scale [38]. Second, floor and ceiling effects were considered problematic if more than 15% of respondents achieved the highest- or lowest-possible score [39, 40]. Third, at the item level, less than 3% missing data was acceptable, whereas more than 15% was not [29].

The reliability of the questionnaire and its subscales were assessed for their internal consistency and stability over time. To assess the internal consistency of the A-items, we used the reliability coefficients of Cronbach’s alpha (α) and composite reliability (ρ_c_), with values ≥.7 considered to be good [29]. Test–retest reliability was evaluated using intra-class correlation coefficients (ICC) to measure the stability of scales over time and weighted kappa values with linear weights for single items [40, 39]. In ICC analysis, a two-way mixed-effect Analysis of Variance (ANOVA) was used because time is a relevant factor in test–retest studies of patient-reported outcome measures. Also, interaction for the absolute agreement between scores was considered the preferred ICC formula [41]. Additionally, measurement error (i.e., standard error of measurement and smallest detectable change) were reported [40].

### Ethical considerations

The Regional Committee for Medical and Health Research Ethics reviewed and approved the study (2009/1225), as did the institutional review board at the three university hospital clinics. Each patient was informed about the study and signed a written declaration of consent to participate. Participants were informed that their participation in the study was voluntary and that they could withdraw their consent at any given time and for any or no reason.

## Results

During the 2-year period of data collection, 360 invitations for participation were sent to eligible patients. At baseline, 208 Norwegian patients with AI completed the questionnaire (57.8% response rate), 50 of whom completed it again after 1–6 weeks (i.e., retest). Observations with one or more missing values across the 17 variables included in any of the three factors were deleted, which left a sample of 161.

At baseline, most respondents were women (87.3%). The age range was 18–89 years (*Mean* 59.2, *SD* = 15.0), as shown in Table 1. Scale scores for the original constructs appear in Table 2.

**Table 1:** Characteristics of participants

Variables	N (%) or mean (SD)	Range
Gender (*n =* 204)		N/A
*women*	178 (87.3)	
*men*	26 (12.7)	
Age (*n =* 193)	59.2 (15.0)	18–89
Hospital (*n =* 208)		N/A
*UNN*	62 (29.8)	
*Ahus*	41 (19.7)	
*St. Olavs*	105 (50.5)	

*UNN* University Hospital of Northern Norway, Tromsø; Ahus = Akershus University Hospital, Oslo; St. Olav’s = St. Olav’s hospital, University hospital, Trondheim. *SD* Standard deviation

**Table 2:** The ICIQ-B original questionnaire including 3 factors and 21 items. Scale means and Standard deviation (SD)

Variable ICIQ-B, 3 factors (***n =*** 161)	Mean (SD)
**1. BOWEL PATTERN (scale score range 1–21)**	
3 On average how many times do you open your bowels in 24 hours?	7.7 (3.12)
4 How often do you open your bowels during the night from going to bed to sleep until you get up in the morning?	
5 Do you have to rush to the toilet when you need to open your bowels?	
6 Do you use medications (tablets or liquids) to stop you opening your bowels?	
7 Do you experience pain/soreness around your back passage?	
**2. BOWEL CONTROL (scale score range 0–28)**	
8 Do you experience any staining of underwear or need to wear pads because of your bowels?	17.3 (5.2)
9 Are you able to control watery or loose stool leaking from your back passage?	
10 Are you able to control accidental loss of formed or solid stool from your back passage?	
11 Are you able to control wind (flatus) escaping from your back passage?	
12 Are you able to control mucus (discharge) leaking from your back passage?	
13 Do you have bowel accidents when you have no need to open your bowels?	
14 Are your bowel accidents or leakages unpredictable?	
**3. QUALITY OF LIFE (scale score range 0–26)**	
19 Do your bowels cause you to feel embarrassed?	17.8 (6.5)
20 Do your bowels cause you to make sure you know where toilets are?	
21 Do your bowels cause you to make plans according to your bowels?	
22 Do your bowels cause you to stay home more often than you would like?	
23 Overall, how much do your bowels interfere with your everyday life?	
**Other bowel symptoms and sexual impact (unscored items):**	
15 Using the pictures please indicate how your bowel movements are most of the time?	N/A
16 Do you need to strain to open your bowels?	
17 Is the possibility of having a bowel accident on your mind?	
18 Do you restrict your sexual activities because of your bowels?	

### Exploratory factor analysis (EFA)

To explain as much of the total variance as possible with as few factors as possible, we subjected the ICIQ-B to EFA. The Kaiser–Meyer–Olkin measure of sampling adequacy, .88, exceeded the recommended value of .60, and Bartlett’s test of sphericity showed statistical significance (*p* < .0001), which supported the factorability of the correlation matrix. A factor loading of .32 indicates approximately 10% overlapping variance with the factor’s other items; thus, a minimum loading of .32 is considered acceptable [42]. Accordingly, a cross-loading item would load at .32 or higher on two or more factors. When subjecting the ICIQ-B to EFA, we sought the cleanest factor structure. Because the original ICIQ-B contains three factors, we expected a three-dimensional structure with correlated factors.

**Fig. 2:**
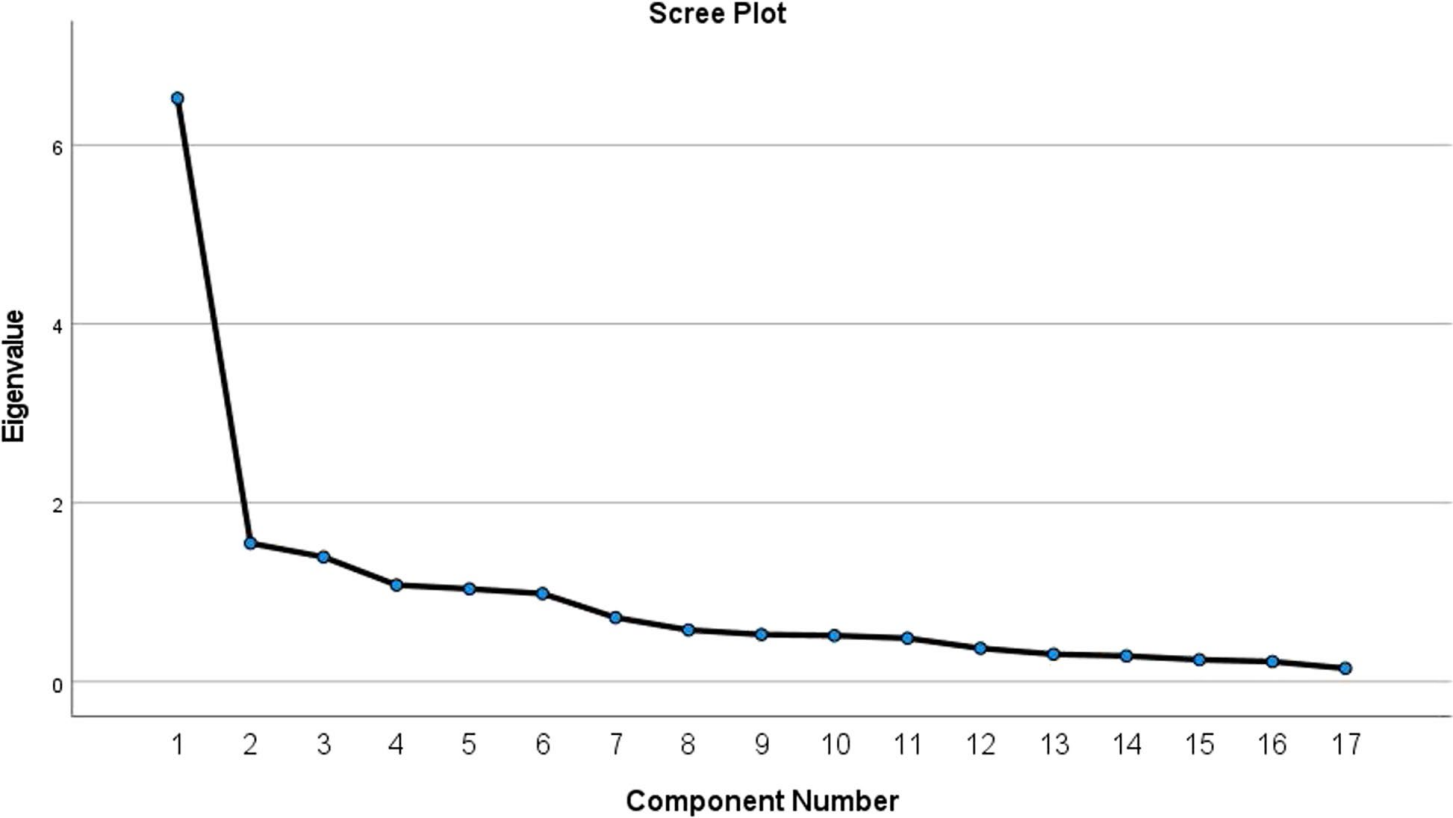
Scree-plot of the 17 item ICIQ-B. Principal component analysis. *N =* 161

Five factors with eigenvalues greater than or equal to 1.0 were extracted (see Table 3), with factor loadings of .38–.94. Figure 2 shows the scree-test of the ICIQ-B data, with five factors explaining 68.17% of the variance; Factor 1 explained 38.37%, Factor 2 explained 9.11%, Factor 3 explained 8.21%, Factor 4 explained 6.37%, and Factor 5 explained 6.11%. That EFA-suggested solution revealed five factors with two to five items each. Four of the factors displayed good or acceptable Cronbach’s alpha coefficients between .64 and .85, whereas the other had a poor one (α = .55). Table 3 lists the loadings and variance for that rotated five-factor solution of the ICIQ-B. Commonalities for the 17 items ranged between .25 for Item 7 and .86 for Item 21, for which a value greater than .40 is recommended [43].

**Table 3:**
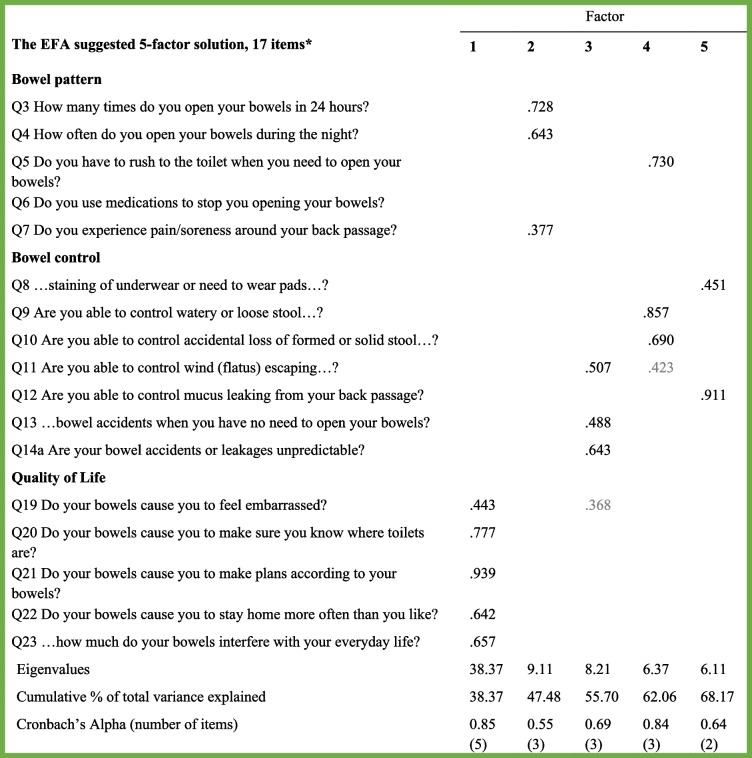
Principal Axis Factoring with oblim rotation of ICIQ-B. Estimates for factor loadings, extraction sums of squared loadings and Cronbach’s alpha

*Note:* ^a^Presented by the original 3 factor solution. All loadings > .4 is included in the table. Loadings are faded if there are higher loadings for the same item. *N =* 161

### Confirmatory factor analysis (CFA)

First, we tested the original three-dimensional structure involving 17 items following Cotterill et al. [18]. The model (i.e., Model 1) revealed standardised factor loadings (λ) of .26–.89, with squared multiple correlations (*R* ^2^) of .007–.79. The fit was poor: Satorra–Bentler χ^2^ = 283.339, *df =* 116, χ^2^/*df =* 2.44, *p = .*0001, RMSEA = .095, *p* for test of close fit = .0001, CFI = .85, TLI = .83, SRMR = .080. Although the estimated χ^2^ value was good, the other fit indices indicated misspecification. Reliability assessed with the composite reliability coefficient (ρ_c_) was good for two of the three dimensions (see Table 4). Analysing the residuals and modification indices (MI) revealed no significant residuals, but 10 MIs were greater than 10; the pairs of Items 11 and 13 (MI = 23.01), Items 3 and 4 (MI = 22.67) and Items 8 and 12 (MI = 19.21) had the highest MIs. Examining the factor loading and *R* ^2^ values revealed that Item 13—“Do you have bowel accidents when you have no need to open your bowels?”—showed a low loading and *R* ^2^ (.09), which suggests less reliability than Item 11—“Are you able to control wind (flatus) escaping from your back passage?” (*R* ^2^ = .42). Because the respondents seemed to regard Item 13 as being irrelevant, we dismissed the item and ran the CFA again. That solution (i.e., Model 2), including 16 items, showed an improved but nevertheless poor fit: Satorra–Bentler χ^2^ = 240.317, *df =* 101, χ^2^/*df =* 2.38, *p = .*0001, RMSEA = .093, *p* for test of close fit = .0001, CFI = .88, TLI = .85, SRMR = .078. Model 2 had seven MIs higher than 10, with Items 3 and 4 presenting the highest value. Again, guided by the factor loadings, *R* ^2^ values and the nuances of the construct, Item 4—“How often do you open your bowels during the night from going to bed to sleep until you get up in the morning?”—showed exceptionally high modification indices with Item 3—“How many times do you open your bowels in 24 hours?”—thereby signifying that the items shared error variance, which makes sense: opening your bowels at night obviously correlates with opening them in the past 24 h. Considering that information regarding the past 24 h was more inclusive than the frequency of opening one’s bowels at night, we kept Item 3 and excluded Item 4 to achieve a statistically good model fit. That solution, Model 3, included 15 of the original items: Satorra–Bentler χ^2^ = 204.662, *df =* 87, χ^2^/*df =* 2.38, *p = .*0001, RMSEA = .092, *p* for test of close fit = .0001, CFI = .89, TLI = .87, SRMR = .073.

**Table 4:** Goodness-of-fit measures for ICIQ-B measurement model. Confirmatory Factor Analysis for Model-1 to Model-8

Fit Measure	Model-1 3-factors 17 items	Model-2 3 factors 16 items	Model-3 3 factors 15 items	Model-4 3 factors 14 items	Model-5 3 factors 13 items	Model-6 3 factors 13 items	Model-7 2 factors 11 items	Model-8 2 factors 10 items
χ^2^	283.339	240.317	204.662	174,77	121,430	108.492	88.725	63.443
*p*-value	0.0001	0.0001	0.0001	0.0001	0.0001	0.0001	0.0001	0.0001
$\frac{x^2}{df}.$	2.44 (Df^1^ = 116)	2.38 (Df^1^ = 101)	2.38 (Df^1^ = 87)	2.36 (Df^1^ = 74)	1.96 (Df^1^ = 62)	1.75 (Df^1^ = 62)	2.06 (Df^1^ = 43)	1.87 (Df^1^ = 34)
RMSEA	0.095	0.093	0.092	0.092	0.077	0.071	0.082	0.074
p-value (close fit test)	0.0001	0.0001	0.0001	0.0001	0.011	0.054	0.003	0.026
SRMR	0.080	0.078	0.073	0.073	0.062	0.056	0.060	0.052
CFI	0.85	0.88	0.89	0.90	0.94	0.95	0.95	0.97
TLI	0.83	0.85	0.87	0.88	0.924	0.94	0.94	0.96
$\textrm{pc}=\frac{{\left(\sum \lambda \right)}^2}{\left[{\left(\sum \lambda \right)}^2+\sum \left(\uptheta \right)\right]}$	0.58–0.89	0.57–0.89	0.52–0.89	0.52–0.89	0.52–0.89	0.51–0.89	0.80, 0.89	0.82, 0.89

Note. ICIQ*-B* International Consultation on Incontinence Questionnaire-Bowels module, *RMSEA* Root Mean Square Error of Approximation, *SRMS* Standardized Root Mean Square Residual, *CFI* The Comparative Fit Index, *TLI* Tucker-Lewis Index, ^1^Df = Degrees of freedom, ρc = Composite reliability, Raykov’s factor reliability coefficient. **Model-1:** 17 items 3-factor solution involving all 17 original items. **Model-2**: 16-items 3-factor solution (item 13 is dismissed). **Model-3**: 15 items 3-factor solution (items 13 and 4 are dismissed). **Model-4**: 14-items 3-factor solution (items 13, 4 and 12 are dismissed). **Model-5**: 13-items 3-factor solution (items 13, 4, 12 and 14 are dismissed). **Model-6**: 13-items 3-factor solution (items 13, 4, 12 and 14 are dismissed and 5 is moved to Control). **Model-7**: 11 items 2-factor solution (Pattern factor and items 13 and 14 are dismissed). **Model-8**: 10 items 2-factor solution (Pattern factor and items 12, 13 and 14 were dismissed). Dismissed items in parenthesis. Listwise *N* = 161

Thus far, we had dismissed Items 13 and 4. Nevertheless, though the χ^2^/*df* was good, the fit remained poor, and five MIs greater than 10 were present. Items 8 and 12 had an MI of 19.43; Item 12—“Are you able to control mucus (discharge) leaking from your back passage?”—shared a considerable amount of error variance with Item 8 (i.e., “Do you experience any staining of your underwear or need to wear pads because of your bowels?”). Because controlling mucus leakage and staining one’s underwear due to such leakage obviously correlate strongly, we dismissed Item 12 to achieve a good fit without including correlated error terms. Nevertheless, that solution (i.e., Model 4), including 14 items, still revealed a poor fit (χ^2^ = 174.77, *df =* 74, χ^2^/*df =* 2.36, *p = .*0001, RMSEA = .092, *p* for test of close fit = .0001, CFI = .90, TLI = .88, SRMR = .073). Furthermore, Items 5 and 14 had an MI of 18 and 16, respectively. The theoretical content of Item 14—“Are your bowel accidents or leakages unpredictable?”—concerned bowel leakage and shared error variance with Item 19—“Do your bowels cause you to feel embarrassed?—which is plausible: bowel accidents and leakage would cause embarrassment. Consequently, removing Item 14 improved the fit.

Even with Item 14 removed, Model 5, including 13 items, only marginally improved the fit. Item 5—“Do you have to rush to the toilet when you need to open your bowels?”—seemed to load more strongly on the Bowel Control factor (λ = 0.82). While allowing it to load on the Bowel Control -factor instead of the Bowel Pattern factor in Model 6 (with 13 items), improved the model fit considerably: χ^2^ = 108,492, *df =* 62, χ^2^/*df =* 1.75, *p = .*0001, RMSEA = .071, *p* for test of close fit = .054, CFI = .95, TLI = .94, SRMR = .056.

As a result, the Bowel Pattern factor, including only three items—Item 3 (i.e. “On average how many times do you open your bowels in 24 hours?”), Item 6 (i.e. “Do you use medications such as tablets or liquids to stop your bowels from opening?”) and Item 7 (i.e. “Do you experience pain/soreness around your back passage?”)—had a low composite reliability (ρ_pattern_ = .51). However, the reliability was good for the other two factors (ρ_control_ = .82 and ρ_QoL_ = .89). Examining the theoretical content of the items belonging to the Bowel Pattern factor clarified that they address different aspects, which explains their low internal consistency. Those items include aspects ranging from the frequency of opening one’s bowels to using medication and experiencing pain. Apparently, the items neither shared much variance nor seemed to represent reliable indicators for the same construct.

Therefore, we dismissed the Bowel Pattern factor (i.e. Items 3, 4, 6 and 7) and re-added Item 12 to the Bowel Control factor. The resulting two-factor solution (i.e., Model 7), with 11 items, revealed a nearly acceptable fit to the data: χ^2^ = 88.725, *df =* 43, χ^2^/*df =* 2.06, *p = .*0001, RMSEA = .082, *p* for test of close fit = .003, CFI = .95, TLI = .94, SRMR = .060. For this model termed Model 7, the loadings ranged between .41 and .90, *R* ^2^ values were between .17 and .81, and composite reliability (ρ_c_) was .80 and .89 for the Bowel Control and Impact on QoL factors, respectively. After again adapting the model, we generated a two-factor model, Model 8, that included 10 of the 17 original items—Items 12–14 along with the Bowel Pattern factor were dismissed—and showed a good fit: χ^2^ = 63.443, *df =* 34, χ^2^/*df =* 1.87, *p = .*0001, RMSEA = .074, *p* for test of close fit = .026, CFI = .97, TLI = .96, SRMR = .052. Composite reliability was .82 and .89 (see Table 5).

**Table 5:** ICIQ-B Model-6 and Model-8 (in parentheses): the best fitting three-factor and two-factor measurement models

Items	Parameter	Stata Estimate	t-value	R^**2**^
**ICIQ-B Pattern**				
ICIQ-B3	λx 1,1	0.49 (−)	5.95^a^	0.24 (−)
ICIQ-B6	λx 2,1	0.69 (−)	7.64^a^	0.48 (−)
ICIQ-B7	λx 3,1	0.27 (−)	2.97^b^	0.07 (−)
**ICIQ-B Control**				
ICIQ-B5	*λx 4,2*	0.82 (0.82)	27.16^a^	0.67 (0.67)
ICIQ-B8	*λx 5,2*	0.44 (0.43)	5.66^a^	0.19 (0.19)
ICIQ-B9	*λx 6,2*	0.82 (0.81)	22.91^a^	0.67 (0.67)
ICIQ-B10	*λx 7,2*	0.76 (0.76)	21.19^a^	0.58 (0.58)
ICIQ-B11	*λx 8,2*	0.58 (0.58)	10.66^a^	0.34 (0.34)
**ICIQ-B Quality of Life (QoL)**				
ICIQ-B19	λx *9,3*	0.57 (0.57)	9.53^a^	0.32 (0.32)
ICIQ-B20	λx *10,3*	0.90 (0.90)	43.12^a^	0.81 (0.81)
ICIQ-B21	λx *11,3*	0.90 (0.90)	48.92^a^	0.81 (0.81)
ICIQ-B22	λx *12,3*	0.81 (0.80)	24.46^a^	0.66 (0,66)
ICIQ-B23	λx *13,3*	0.83 (0.83)	28.78^a^	0.69 (0.69)
*ρ*_*c*_Pattern	*ρ*_*c*_	0.51 (−)		
*ρ*_*c*_Control	*ρ*_*c*_	0.80 (0.82)		
*ρ*_*c*_Quality of life	*ρ*_*c*_	0.89 (0.89)		

*Note.* Model-6: three-factor solution including 13 items (items 13,4,12 and 14 are dismissed and item 5 is moved to Control). Model-8: Two-factor solution including factors ICIQ-B Control and ICIQ-B Quality of Life and 10 items (items 12–14 are dismissed); estimates for Model-8 are in parenthesis. ^a^Significant at the 1% level, ^b^ Significant at the 5% level. Completely Standardized Factor Loadings. Bentler-Raykov squared multiple correlation coefficient = R^2^. Listwise, *N =* 161, Composite reliability 𝝆C = $\frac{{\left(\sum \lambda \right)}^2}{{\left(\sum \lambda \right)}^2+\sum \left(\uptheta \right)}$

**Fig. 3:**
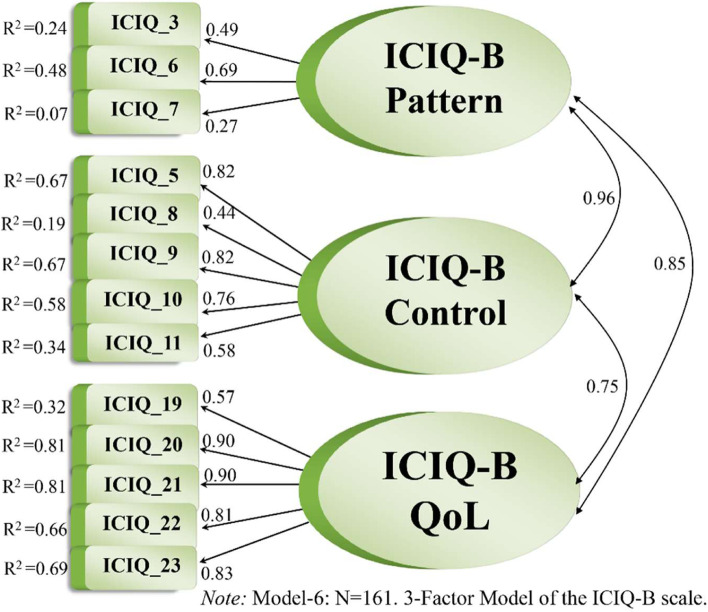
The best fitting most parsimonious measurement model of the Norwegian version ICIQ-B scale

**Fig. 4:**
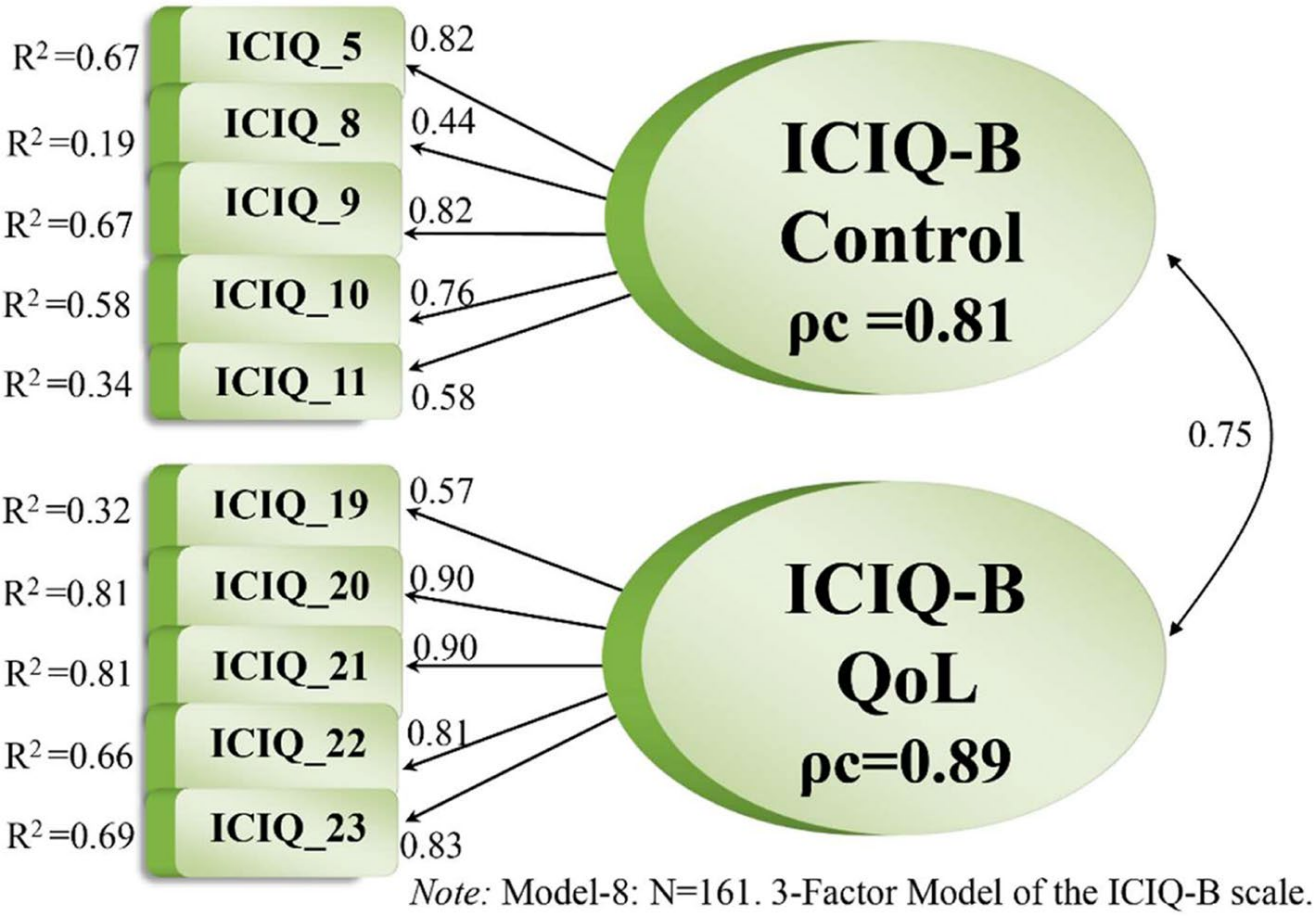
The best fitting two-factor solution of the Norwegian version of the ICIQ-B scale

Thus, Model 8, with two factors (i.e. Bowel Control and Impact on QoL) and 10 items, was less parsimonious but demonstrated the statistically best fit. By comparison, Model 6 also included those factors along with the Bowel Pattern factor, with 13 items, and was therefore the most parsimonious measurement model with a good fit. Models 6 and 8 are illustrated in Figs. 3 and 4, respectively.

### Content validity and scale reliability

Cognitive interviews with patients with AI and evaluations of the Norwegian ICIQ-B version by clinical experts indicated the Norwegian ICIQ-B’s good face and content validity in terms of relevance, comprehensiveness, readability, and equivalence. The overall percentage of missing data at baseline was 3.3%, ranging from 0.5 to 11.1% for single items. Because none of the proposed scales had the lowest- or highest-possible score with more than 15% frequency, no floor or ceiling effects were found in the total score distributions.

Regarding reliability, internal consistency in the proposed factors showed Cronbach’s alphas (α) from .37 to .85, as presented in Table 6. Test–retest stability revealed ICCs between .90 and .94. Concerning the stability of single items, 13 items had weighted kappa values of .61–.80, whereas five had values of .41–.60, two of .81–1.00 and one of .21–.40. The factors’ standard error of measurement error (*SEM*), an expression of the average measurement error, was estimated to be 0.42–0.73 points, while the smallest detectable change (SDC^95^), indicating the uncertainty of that average, was 1.16–2.02 points [29]. Although the *SEM* was 0.73 for the factor Impact on QoL, to be 95% certain that a change beyond the measurement error has occurred, the patient’s score has to change by 2.02 points from test to retest.

**Table 6:** Weighted Kappa, ICC, Cronbach’s alpha and change score for new subscales, Bristol stool chart and single items in the Norwegian version of ICIQ-B scale

	Reliability			Change score		
	Weighted Kappa (95% CI) *n =* 50	ICC^a^ (95% CI) *n =* 50	Cronbach’s alpha (*n =* 161)	Mean (SD) (*n =* 50)	SEM^b^	SDC^c^
Impact on QoL						
Q19	0.65 (0.46–0.83)	0.94 (0.90–0.97) *n =* 41	0.85	0.46 (2.97) *n =* 41	0.73	2.02
Q20	0.72 (0.46–0.83)					
Q21	0.63 (0.48–0.78)					
Q22	0.73 (0.59–0.86)					
Q23	0.54 (0.39–0.69)					
Control						
Q5	0.57 (0.42–0.72)	0.92 (0.85–0.95) *n =* 44	0.80	0.49 (2.41) *n =* 45	0.68	1.88
Q8	0.63 (0.49–0.78)					
Q9	0.73 (0.60–0.86)					
Q10	0.65 (0.51–0.80)					
Q11	0.68 (0.55–0.82)					
Pattern						
Q3	0.58 (0.34–0.81)	0.90 (0.81–0.94) *n =* 45	0.37	−0.13 (1.32) *n =* 45	0.42	1.16
Q6	0.76 (0.42–0.72)					
Q7	0.64 (0.47–0.80)					
Bristol stool chart						
Q15 -Bristol stool chart	0.87 (0.73–1.01)					
Single unscored items – outside the factor structure						
Q4	0.48 (0.24–0.73)					
Q12	0.57 (0.41–0.72)					
Q13	0.42 (0.23–0.61)					
Q14	0.39 (0.17–0.60)					
Q16	0.65 (0.48–0.82)					
Q17	0.61 (0.46–0.77)					
Q18	0.67					

Note: ^a^ICC two-way mixed, absolute agreement, average measure. ^b^ $\textrm{SEM}=\textrm{SD}\sqrt{1-\textrm{ICC}}$

^c^ *SDC* ^*95*^ Smallest Detectable Change with 95% certainty =1.96 x $\sqrt{2}\ \textrm{x}\ \textrm{SEM}.$

## Discussion

The original ICIQ-B includes 17 items representing three factors (i.e. Bowel Pattern, Bowel Control, and Impact on QoL), along with four unscored items. In our study, we translated the ICIQ-B scale into Norwegian and tested its psychometric properties (i.e. structural validity, reliability, and content validity) amongst adults in Norway.

### Structural validity

When evaluating a measurement scale’s structural validity, two aspects are vital: the data’s underlying dimensionality (i.e., not too many or too few factors) and the adequacy of the scale’s individual items [29]. Showing eigenvalues exceeding 1.0, our EFA suggested five factors: two substantial factors with three and five items and three weak factors with three or two items each. The EFA also revealed cross-loadings, and because the original ICIQ-B contains only three factors [18], its dimensionality seemed uncertain. However, because conclusions should not be drawn solely based on EFA, we conducted a CFA, which revealed both a three-factor solution and a two-factor measurement model showing good fit. However, several items seemed to indicate misspecification.

Both reliability and structural validity relate to the adequacy of a scale’s items. Good indicators of a factor show highly significant factor loadings, preferably greater than .70, accompanied by strong squared multiple correlations (*R* ^2^), which represent how much variation in an item is explained by the latent construct [44]. In our study, all loadings were significant at the 1% level except Item 7. Regarding Model 6, Table 5 shows that seven factor loadings were excellent (>.70), four were good to fair (.55–.45), and two, for Items 7 and 8, were very low (<.45) and hardly explained any variance in the respective construct [28]. Thus, 11 items were rated as reliable indicators, whereas Items 7 and 8 displayed poor reliability. For Model 6, the factors of Bowel Control and Impact on QoL had good alpha values and composite reliability(ρ_c_), whereas the Bowel Pattern factor demonstrated low internal consistency (ρ_c_ = .51) and thus low reliability [45, 33]. Accordingly, the dimensionality seemed imprecise, as further pinpointed by Item 5’s far stronger loading on another factor than originally determined. Allowing Item 5 (i.e. “Do you have to rush to the toilet when you need to open your bowels?”) relate to the Bowel Control factor instead of the Bowel Pattern factor (Model 6) improved the model fit considerably. Based on the low reliability of the Bowel Pattern factor, we tested a two-factor solution excluding that factor. That solution (i.e. Model 8) showed good reliability, with highly significant factor loadings, good reliability coefficients and a nearly acceptable fit. Looking at the two-factor model including Item 5 in the Bowel Control factor, the solution had good reliability and clear dimensionality. However, to achieve a good model fit, some items had to be removed, namely Items 12–14, all of which solicit information about bowel accidents. In our study, respondents seemed to assume those three items sought to assess roughly the same thing, which generated substantial correlated error variance that again hampered the model fit.

In our investigation, the original three-factor structure with 17 items did not fit well with the data. Model 6, including three factors and 13 items, was the most parsimonious model with a good fit, whereas Model 8, including two factors with 10 items, was less parsimonious but demonstrated a statistical better fit. Both models contained identical versions of the factors of Bowel Control and Impact on QoL and differed only considering Model 6’s inclusion of the third factor, Bowel Pattern.

### Content validity

To gauge the translated scale’s relevance, comprehensiveness and comprehensibility, cognitive interviews with patients from the target group and evaluations made by a multidisciplinary group of clinical experts deemed that the content and wording of the Norwegian ICIQ-B’s items corresponded well with the constructs intended to be measured—that is, the items captured AI’s complexity [29]. However, the items did not fit well into the three constructs, especially for the construct of bowel pattern, in which the items were overly broad and caused insufficient internal consistency, as also seen in the original British English version, the Spanish version and the American English version [18, 23, 24]. Moreover, the original ICIQ-B includes four unscored items not encompassed within the original dimensionality. The four items (i.e. Items 4 and 12–14) removed from the Norwegian scale, however, could be placed together with those four unscored items, which would support the Norwegian ICIQ-B’s clinical relevance.

The Norwegian ICIQ-B with the adapted factor structure demonstrated promising psychometric properties. The level of missing items in the questionnaire was acceptable, which confirmed that that the items were relevant, straightforward, and meaningful to the respondents. One item had more than 3% missing data, namely Item 18 (i.e. “Do you restrict your sexual activities because of your bowels?”), with 11% missing data. That outcome is unsurprising, because sexuality may be a sensitive topic or even be perceived as irrelevant. The absence of floor and ceiling effects demonstrated that the scale could produce a good distribution of responses to a given item and that scores at the scale’s upper or lower levels show no clustering or skewness. That measurement property is also important regarding the questionnaire’s discriminative power. For example, a maximum score would preclude recognising any potential improvement to the questionnaire following any type of intervention.

### Scale reliability

Testing the Norwegian ICIQ-B demonstrated its good reliability in terms of internal consistency and excellent stability. While the Bowel Control factor had an acceptable Cronbach’s alpha, Impact on QoL factor had a good one. However, for the Bowel Pattern factor (α = .37), the reliability coefficient was unacceptably low (>.5) [46]. The poor reliability of the Bowel Pattern factor has previously been identified, including in the initial study by the scale’s developers [24, 18]. Consistent with the American English and Spanish versions of the ICIQ-B and the initial study performed by the developers [24, 23, 18], stability over time was excellent for all three constructs [47]. Furthermore, the Norwegian ICIQ-B demonstrated stability for 13 single items with largely substantial weighted kappa values, two with nearly perfect values, five with moderate values and one with a fair value [48]. The good test–retest reliability of an instrument ensures that measurements obtained are both representative and stable over time [29].

### Limitations

A major strength of our study was the rigorous methodology employed in translating and validating the Norwegian ICIQ-B following COSMIN guidelines [26]. However, some limitations should be noted. First, the sample size of 208 was scaled down to 161 due to missing data. The response rate was nevertheless sufficient to perform the analysis. Second, this study employed a rather wide time frame between test and retest with a risk for recall bias and changes in the respondent’s health status. Finally, it is worth noting that a good model fit does not guarantee that we have obtained ‘the true model’; other alternative models might fit the data equally well as the model found [49].

## Conclusion

To determine the psychometric properties of the Norwegian ICIQ-B, we assessed the translated scale’s dimensionality, reliability, and content validity. The dimensionality seemed inaccurate. We were able to present a three-factor and a two-factor solution, both with advantages and disadvantages. The three-factor model represents the most parsimonious solution due to covering most of the original scale, albeit with unacceptably low reliability for the Bowel Pattern factor. The two-factor model demonstrates good reliability but is less parsimonious due to lacking seven of the original 17 items and excluded one of the constructs. For a statistically well-functioning measurement model able to be used in SEM or regression analysis, we consider the two-factor construct to be superior. By contrast, concerning the clinical relevance, breadth and nuances of the theoretical constructs, the three-factor solution consisting of 13 items is superior. In addition, the eight unscored and removed items may be used in a clinical context to provide more information about the patient’s condition. The two factors Bowel Control and Impact on QoL are identical in the two models in terms of included items and psychometric properties, meaning that the models differed only in Model 6’s inclusion of the Bowel Pattern factor, which may be used in a clinical context. Altogether, the Norwegian ICIQ-B has excellent reliability in terms of test–retest stability, good internal consistency for the two-factor model and good content validity.

The results recommend further studies of the Norwegian ICIQ-B’s psychometric properties to gain more in-depth clinical insights into improving the reliability and construct validity of the ICIQ-B as a measure of patient-reported outcomes. After all, a single study does not prove structural validity. On the contrary, structural validation is a continuous process of evaluation, re-evaluation, refinement, and development.

## Supplementary Information

**Additional file 1.**

## Data Availability

Due to the sensitive nature of the dataset which the analysis is based upon, it is not publicly available. The present Norwegian legislation and the General Data Protection Regulation (GDPR) of the European Union does not allow sensitive data to be made openly accessible. In special cases data is available from the authors on reasonable request.

## Abbreviations

ICIQ-B: The International Consultation on Incontinence Questionnaire-Bowel
AI: Anal Incontinence
QoL: Quality of Life
FI: Faecal Incontinence
COSMIN: COnsensus-based Standards for the selection of health Measurement Instruments
EFA: Exploratory Factor Analysis
CFA: Confirmatory factor analysis
RMSEA: Root Mean Square Error of Approximation
SRMS: Standardized Root Mean Square Residual
CFI: Comparative Fit Index
TLI: Tucker-Lewis Index
α: Cronbach’s alpha
ρ_c_: Composite Reliability
ICC: Intraclass Correlation Coefficient
ANOVA: Analysis of Variance
UNN: University Hospital of Northern Norway, Tromsø
Ahus: Akershus University Hospital, Oslo
SD: Standard Deviation
SEM: Standard Error of Measurement
SDC: Smallest Detectable Change
R^2^-values: Squared multiple correlations
MI: Modification indices
Df: Degrees of freedom.

## References

[1] Meyer I, Richter HE (2015). Impact of fecal incontinence and its treatment on quality of life in women. Women’s Health.

[2] Norton C, Whitehead WE, Bliss DZ, Harar D, Lang J (2010). Management of fecal incontinence in adults: report from the 4th International Consultation on Incontinence. Neurourol Urodyn.

[3] Robson KM. Fecal incontinence in adults: Etiology and evaluation. UpToDate September 2020. https://www.uptodate.com/contents/fecal-incontinence-in-adults-etiology-and-evaluation?search=defecation&source=search_result&selectedTitle=2~149&usage_type=default&display_rank=2#H9. Accessed 27 Jan 2022.

[4] Sharma A, Yuan L, Marshall RJ, Merrie AE, Bissett IP (2016). Systematic review of the prevalence of faecal incontinence. Br J Surg.

[5] Rømmen K, Schei B, Rydning A, Sultan AH, Mørkved S (2012). Prevalence of anal incontinence among Norwegian women: a cross-sectional study. BMJ Open.

[6] Johanson JF, Lafferty J (1996). Epidemiology of fecal incontinence: the silent affliction. Am J Gastroenterol.

[7] Musa MK, Saga S, Blekken LE, Harris R, Goodman C, Norton C (2019). The Prevalence, Incidence, and Correlates of Fecal Incontinence Among Older People Residing in Care Homes: A Systematic Review. J Am Med Dir Assoc.

[8] Saldana Ruiz N, Kaiser AM (2017). Fecal incontinence - Challenges and solutions. World J Gastroenterol.

[9] Andrews CN, Bharucha AE (2005). The etiology, assessment, and treatment of fecal incontinence. Nat Clin Pract Gastroenterol Hepatol.

[10] Shin GH, Toto EL, Schey R (2015). Pregnancy and postpartum bowel changes: constipation and fecal incontinence. Am J Gastroenterol.

[11] Ternent CA, Fleming F, Welton ML, Buie WD, Steele S, Rafferty J (2015). Clinical Practice Guideline for Ambulatory Anorectal Surgery. Dis Colon Rectum.

[12] Wallenhorst T, Bouguen G, Brochard C, Cunin D, Desfourneaux V, Ropert A, Bretagne JF, Siproudhis L (2015). Long-term impact of full-thickness rectal prolapse treatment on fecal incontinence. Surgery..

[13] Walma MS, Kornmann VN, Boerma D, de Roos MA, van Westreenen HL (2015). Predictors of fecal incontinence and related quality of life after a total mesorectal excision with primary anastomosis for patients with rectal cancer. Ann Coloproctol.

[14] Rao SS (2004). Diagnosis and management of fecal incontinence. American College of Gastroenterology Practice Parameters Committee. Am J Gastroenterol.

[15] Madoff RD (2004). Surgical treatment options for fecal incontinence. Gastroenterology.

[16] Wiebe S, Guyatt G, Weaver B, Matijevic S, Sidwell C (2003). Comparative responsiveness of generic and specific quality-of-life instruments. J Clin Epidemiol.

[17] Cotterill N, Norton C, Avery KN, Abrams P, Donovan JL (2008). A patient-centered approach to developing a comprehensive symptom and quality of life assessment of anal incontinence. Dis Colon Rectum.

[18] Cotterill N, Norton C, Avery KNL, Abrams P, Donovan J (2011). Psychometric Evaluation of a New Patient-Completes Questionnaire for Evaluating Anal Incontinence Symptoms and Impact on Quality of Life: The ICIQ-B. Dis Colon Rectum.

[19] The International Consultation on Incontinence Questionnaire. https://iciq.net/. Accessed 20 Jan 2022.

[20] Lewis SJ, Heaton KW (1997). Stool form scale as a useful guide to intestinal transit time. Scand J Gastroenterol.

[21] Black N (2013). Patient reported outcome measures could help transform healthcare. BMJ..

[22] Basch E (2017). Patient-Reported Outcomes - Harnessing Patients' Voices to Improve Clinical Care. N Engl J Med.

[23] Sacomori C, Lorca LA, Martinez-Mardones M, Benavente P, Plasser J, Pardoe M (2021). Spanish Translation, Face Validity, and Reliability of the ICIQ-B Questionnaire with Colorectal Cancer Patients. J Coloproctol.

[24] Markland AD, Burgio KL, Beasley TM, David SL, Redden DT, Goode PS (2017). Psychometric evaluation of an online and paper accidental bowel leakage questionnaire: The ICIQ-B questionnaire. Neurourol Urodyn.

[25] Lee JT, Madoff RD, Rockwood T (2015). Quality-of-Life Measures in Fecal Incontinence: Is Validation Valid?. Dis Colon & Rectum.

[26] Mokkink LB, Prinsen CAC, Patrick DL, Alonso J, Bouter LM, de Vet HCW, et al. COSMIN Study Design checklist for Patient-reported outcome measurement instruments. COSMIN. 2019; https://www.cosmin.nl/wp-content/uploads/COSMIN-study-designing-checklist_final.pdf. Accessed 7 Jan 2022.

[27] Gagnier JJ, Lai J, Mokkink LB, Terwee CB (2021). COSMIN reporting guideline for studies on measurement properties of patient-reported outcome measures. Qual Life Res.

[28] Netemeyer RG, Bearden WO, Sharma S (2003). Scaling procedures: issues and applications.

[29] de Vet HW, Terwee CB, Mokkink LB, Knol DL (2011). Measurement in medicine – Practical guide to biostatistics and epidemiology.

[30] ICIQ Validation methodology. https://iciq.net/validation-methodology. Accessed 20 Jan 2022.

[31] Pett MA, Lackey NR, Sullivan JJ (2003). Making sense of factor analysis: the use of factor analysis for instrument development in health care research.

[32] StataCorp. (2021). Stata Statistical Software: Release 17.

[33] Mehmetoglu M, Jakobsen TG (2017). Applied Statistics using STATA.

[34] McCallum RC, Austin JT (2000). Applications of structural equation modeling in psychological research. Annu Rev Psychol.

[35] Acock AC (2013). Discovering structural equation modeling using Stata.

[36] Schermelleh-Engel K, Moosbrugger H, Müller H (2003). Evaluating the Fit of Structural Equation Models: Tests of Significance and Descriptive Goodness-of-Fit. Measures. Methods of Psychol Res.

[37] Kline RB (2011). Principles and practice of structural equation modeling.

[38] Willis GB. Cognitive Interviewing - A “How To” Guide. Short course presented at the 1999 Meeting of the American Statistical Association. https://www.hkr.se/contentassets/9ed7b1b3997e4bf4baa8d4eceed5cd87/gordonwillis.pdf. Accessed 19 Apr 2021.

[39] Mokkink LB, Terwee CB, Patrick DL, Alonso J, Stratford PW, Knol DL, Bouter LM, de Vet HC (2010). The COSMIN checklist for assessing the methodological quality of studies on measurement properties of health status measurement instruments: an international Delphi study. Qual Life Res.

[40] Terwee CB, Bot SD, de Boer MR, van der Windt DA, Knol DL, Dekker J, Bouter LM, de Vet HC (2007). Quality criteria were proposed for measurement properties of health status questionnaires. J Clin Epidemiol.

[41] Qin S, Nelson L, McLeod L, Eremenco S, Coons SJ (2019). Assessing test-retest reliability of patient-reported outcome measures using intraclass correlation coefficients: recommendations for selecting and documenting the analytical formula. Qual Life Res.

[42] Tabachnick BG, Fidell LS (2013). Using multivariate statistics.

[43] Osborne JW, Costello AB, Kellow JT, Osborne JW (2008). Best Practices in exploratory factor analysis. Best Practices in Quantitative Methods.

[44] Raykov T (2001). Estimation of congeneric scale reliability using covariance structure analysis with nonlinear constraints. Br J Math Stat Psychol.

[45] Hair JJ, Black W, Babin B, Anderson R (2010). Multivariate data analysis.

[46] George D, Mallery P (2003). SPSS for Windows step by step: A simple guide and reference. 11.0 update.

[47] Koo TK, Li MY (2016). A guideline of selecting and reporting intraclass correlation coefficients for reliability research. J Chiropr Med.

[48] Landis JR, Koch GC (1977). The measurement of observer agreement for categorical data. Biometrics..

[49] Bollen KA (1989). Structural equations with latent variables.

