# Supplementary material for: Symptoms of anal incontinence and quality of life: a psychometric study of the Norwegian version of the ICIQ-B amongst hospital outpatients
Source: Arch Public Health. 2022 Dec 9;80:251. doi: 10.1186/s13690-022-01004-z (PMC9733285; doi:10.1186/s13690-022-01004-z)
Supplement: Supplementary file 1 — Additional file 1. [file 13690_2022_1004_MOESM1_ESM.pdf]

Konfidensielt

Dato for utfylling: (dd.mm.yyyy) ☐☐.☐☐.☐☐☐☐

Mange mennesker opplever å ha avføringsuhell eller lekkasjer fra endetarmen. Vi prøver å finne ut hvor mange som opplever disse symptomene og hvor mye det plager dem. Vi vil sette pris på om du kan svare på spørsmålene som følger, med tanke på hvordan du har hatt det DE SISTE TRE MÅNEDENE.

1 Fødselsår ☐☐☐☐

2 Kjønn ☐ Kvinne ☐ Mann

Avføringsmønster

3 Hvor mange ganger har du vanligvis avføring i løpet av 24 timer?

Kryss av ett svaralternativ for "vanligvis", og kryss av ett svaralternativ for "på det verste".

	(a) Vanligvis	(b) På det verste
Mindre enn én gang	☐ 1	☐ 1
En til tre ganger	☐ 2	☐ 2
Tre til ti ganger	☐ 3	☐ 3
Ti eller flere ganger	☐ 4	☐ 4

(c) **Hvor mye plager dette deg?**
Sett kryss ved et tall fra 0 *(ikke i det hele tatt)* til 10 *(svært mye)*

☐ 0 ☐ 1 ☐ 2 ☐ 3 ☐ 4 ☐ 5 ☐ 6 ☐ 7 ☐ 8 ☐ 9 ☐ 10

ikke i det hele tatt **svært mye**

4 Hvor ofte har du avføring i løpet av natten fra du legger deg og til du står opp om morgenen? *(Kryss av ett svaralternativ)*

Aldri	☐ 0
En gang	☐ 1
To ganger	☐ 2
Tre ganger	☐ 3
Fire eller flere ganger	☐ 4

(b) **Hvor mye plager dette deg?**
Sett kryss ved et tall mellom 0 *(ikke i det hele tatt)* og 10 *(svært mye)*

☐ 0 ☐ 1 ☐ 2 ☐ 3 ☐ 4 ☐ 5 ☐ 6 ☐ 7 ☐ 8 ☐ 9 ☐ 10

ikke i det hele tatt **svært mye**

5 Må du skynde deg på toalettet når du skal ha avføring? *(Kryss av ett svaralternativ)*

(a)

Aldri ☐ 0
Sjelden ☐ 1
Av og til ☐ 2
Vanligvis ☐ 3
Alltid ☐ 4

(b) Hvor mye plager dette deg?
Sett kryss ved et tall mellom 0 *(ikke i det hele tatt)* og 10 *(svært mye)*

☐ 0 ☐ 1 ☐ 2 ☐ 3 ☐ 4 ☐ 5 ☐ 6 ☐ 7 ☐ 8 ☐ 9 ☐ 10

ikke i det hele tatt **svært mye**

6 Bruker du medikamenter (tabletter eller miksturer) for å <u>stoppe</u> avføringen?
(Kryss av ett svaralternativ)

(a)

Aldri ☐ 0
Sjeldnere enn en gang i måneden ☐ 1
Sjeldnere enn en gang i uka ☐ 2
Sjeldnere enn en gang om dagen ☐ 3
Omtrent en gang om dagen ☐ 4
Flere ganger daglig ☐ 5

(b) Hvor mye plager dette deg?
Sett kryss ved et tall mellom 0 *(ikke i det hele tatt)* og 10 *(svært mye)*

☐ 0 ☐ 1 ☐ 2 ☐ 3 ☐ 4 ☐ 5 ☐ 6 ☐ 7 ☐ 8 ☐ 9 ☐ 10

ikke i det hele tatt **svært mye**

7 Har du smerter eller svie rundt endetarmen?
(Kryss av ett svaralternativ)

(a)

Aldri ☐ 0
Sjelden ☐ 1
Av og til ☐ 2
Vanligvis ☐ 3
Alltid ☐ 4

(b) Hvor mye plager dette deg?
Sett kryss ved et tall mellom 0 *(ikke i det hele tatt)* og 10 *(svært mye)*

☐ 0 ☐ 1 ☐ 2 ☐ 3 ☐ 4 ☐ 5 ☐ 6 ☐ 7 ☐ 8 ☐ 9 ☐ 10

ikke i det hele tatt **svært mye**

Avføringsmønster: sum skår 3a - 7a ☐☐

Tarmkontroll

8 Opplever du å ha avføringsflekker i undertøyet ditt eller at du må bruke innlegg på grunn av lekkasje? *(Kryss av ett svaralternativ)*

	(a)
Aldri	☐ 0
Sjeldnere enn en gang i måneden	☐ 1
Sjeldnere enn en gang i uka	☐ 2
Sjeldnere enn en gang om dagen	☐ 3
Hver dag	☐ 4

(b) Hvor mye plager dette deg?
Sett kryss ved et tall mellom 0 *(ikke i det hele tatt)* og 10 *(svært mye)*

☐ 0 ☐ 1 ☐ 2 ☐ 3 ☐ 4 ☐ 5 ☐ 6 ☐ 7 ☐ 8 ☐ 9 ☐ 10

ikke i det hele tatt **svært mye**

9 Klarer du å holde på flytende eller løs avføring?
(Kryss av ett svaralternativ)

	(a)
Alltid	☐ 0
Vanligvis	☐ 1
Av og til	☐ 2
Sjelden	☐ 3
Aldri	☐ 4

(b) Hvor mye plager dette deg?
Sett kryss ved et tall mellom 0 *(ikke i det hele tatt)* og 10 *(svært mye)*

☐ 0 ☐ 1 ☐ 2 ☐ 3 ☐ 4 ☐ 5 ☐ 6 ☐ 7 ☐ 8 ☐ 9 ☐ 10

ikke i det hele tatt **svært mye**

10 Klarer du å holde på fast eller hard avføring?
(Kryss av ett svaralternativ)

	(a)
Alltid	☐ 0
Vanligvis	☐ 1
Av og til	☐ 2
Sjelden	☐ 3
Aldri	☐ 4

(b) Hvor mye plager dette deg?
Sett kryss ved et tall mellom 0 *(ikke i det hele tatt)* og 10 *(svært mye)*

☐ 0 ☐ 1 ☐ 2 ☐ 3 ☐ 4 ☐ 5 ☐ 6 ☐ 7 ☐ 8 ☐ 9 ☐ 10

ikke i det hele tatt **svært mye**

11 Klarer du å holde på luft (promp)?
(Kryss av ett svaralternativ)

(a)

Alltid	☐ 0
Vanligvis	☐ 1
Av og til	☐ 2
Sjelden	☐ 3
Aldri	☐ 4

(b) Hvor mye plager dette deg?
Sett kryss ved et tall mellom 0 *(ikke i det hele tatt)* og 10 *(svært mye)*

☐ 0 ☐ 1 ☐ 2 ☐ 3 ☐ 4 ☐ 5 ☐ 6 ☐ 7 ☐ 8 ☐ 9 ☐ 10

ikke i det hele tatt **svært mye**

12 Klarer du å holde igjen siv av slim fra endetarmen?
(Kryss av ett svaralternativ)

(a)

Alltid	☐ 0
Vanligvis	☐ 1
Av og til	☐ 2
Sjelden	☐ 3
Aldri	☐ 4

(b) Hvor mye plager dette deg?
Sett kryss ved et tall mellom 0 *(ikke i det hele tatt)* og 10 *(svært mye)*

☐ 0 ☐ 1 ☐ 2 ☐ 3 ☐ 4 ☐ 5 ☐ 6 ☐ 7 ☐ 8 ☐ 9 ☐ 10

ikke i det hele tatt **svært mye**

13 Har du luft- eller avføringsuhell uten at du føler trang til å gå på toalettet?
(Kryss av ett svaralternativ)

(a)

Aldri	☐ 0
Sjelden	☐ 1
Av og til	☐ 2
Vanligvis	☐ 3
Alltid	☐ 4

(b) Hvor mye plager dette deg?
Sett kryss ved et tall mellom 0 *(ikke i det hele tatt)* og 10 *(svært mye)*

☐ 0 ☐ 1 ☐ 2 ☐ 3 ☐ 4 ☐ 5 ☐ 6 ☐ 7 ☐ 8 ☐ 9 ☐ 10

ikke i det hele tatt **svært mye**

14 Er dine uhell/ lekkasjer i forhold til avføring/ luft uforutsigbare?
(Kryss av ett svaralternativ)

(a)

Aldri ☐ 0
Sjelden ☐ 1
Av og til ☐ 2
Vanligvis ☐ 3
Alltid ☐ 4

(b) Hvor mye plager dette deg?
Sett kryss ved et tall mellom 0 *(ikke i det hele tatt)* og 10 *(svært mye)*

☐ 0 ☐ 1 ☐ 2 ☐ 3 ☐ 4 ☐ 5 ☐ 6 ☐ 7 ☐ 8 ☐ 9 ☐ 10

ikke i det hele tatt **svært mye**

Tarmkontrollskår: sum skår 8a - 14a

Andre luft- og avføringssymptomer

15 Bruk bildene og kryss av for hvordan din avføring som oftest ser ut.
(Sett kryss ved alle bildene som passer)

(a)

Harde, separate knoller, som nøtter (vanskelig å trykke ut) 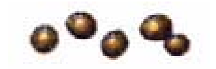 ☐ 1
Pølseformet, med klumper 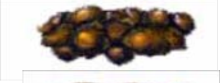 ☐ 2
Pølseformet, med sprekker på overflaten 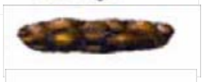 ☐ 3
Pølseformet eller slangeformet - glatt og myk 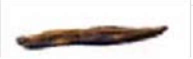 ☐ 4
Myke klumper, med klar avgrensning (lett å trykke ut) 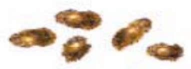 ☐ 5
Luftige klatter som er frynsete i kantene, en grøtete avføring 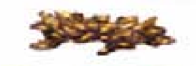 ☐ 6
Flytende, ingen faste elementer 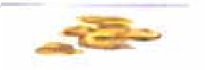 ☐ 7

(b) Hvor mye plager dette deg?
Sett kryss ved et tall mellom 0 *(ikke i det hele tatt)* og 10 *(svært mye)*

☐ 0 ☐ 1 ☐ 2 ☐ 3 ☐ 4 ☐ 5 ☐ 6 ☐ 7 ☐ 8 ☐ 9 ☐ 10

ikke i det hele tatt **svært mye**

16 Må du presse for å ha avføring? *(Kryss av ett svaralternativ)*

	(a)
Aldri	☐ 0
Sjelden	☐ 1
Av og til	☐ 2
Vanligvis	☐ 3
Alltid	☐ 4

(b) Hvor mye plager dette deg?
Sett kryss ved et tall mellom 0 *(ikke i det hele tatt)* og 10 *(svært mye)*

☐ 0 ☐ 1 ☐ 2 ☐ 3 ☐ 4 ☐ 5 ☐ 6 ☐ 7 ☐ 8 ☐ 9 ☐ 10

ikke i det hele tatt **svært mye**

17 Bekymrer du deg over mulige luft- og avføringsuhell?

	(a)
Aldri	☐ 0
Sjelden	☐ 1
Av og til	☐ 2
Vanligvis	☐ 3
Alltid	☐ 4

(b) Hvor mye plager dette deg?
Sett kryss ved et tall mellom 0 *(ikke i det hele tatt)* og 10 *(svært mye)*

☐ 0 ☐ 1 ☐ 2 ☐ 3 ☐ 4 ☐ 5 ☐ 6 ☐ 7 ☐ 8 ☐ 9 ☐ 10

ikke i det hele tatt **svært mye**

Innvirkning på seksuallivet

18 Begrenser du ditt seksualliv på grunn av mulige uhell/ lekkasjer i forhold til avføring/luft? *(Kryss av ett svaralternativ)*

	(a)
Aldri	☐ 0
Sjelden	☐ 1
Av og til	☐ 2
Vanligvis	☐ 3
Alltid	☐ 4
Ikke aktuelt	☐ 5

(b) Hvor mye plager dette deg?
Sett kryss ved et tall mellom 0 *(ikke i det hele tatt)* og 10 *(svært mye)*

☐ 0 ☐ 1 ☐ 2 ☐ 3 ☐ 4 ☐ 5 ☐ 6 ☐ 7 ☐ 8 ☐ 9 ☐ 10

ikke i det hele tatt **svært mye**

Livskvalitet

19 Gjør dine uhell/ lekkasjer i forhold til avføring/luft deg flau? *(Kryss av ett svaralternativ)*

(a)

Aldri	☐ 0
Sjelden	☐ 1
Av og til	☐ 2
Vanligvis	☐ 3
Alltid	☐ 4

(b) Hvor mye plager dette deg?
Sett kryss ved et tall mellom 0 *(ikke i det hele tatt)* og 10 *(svært mye)*

☐ 0 ☐ 1 ☐ 2 ☐ 3 ☐ 4 ☐ 5 ☐ 6 ☐ 7 ☐ 8 ☐ 9 ☐ 10

ikke i det hele tatt **svært mye**

20 Gjør dine uhell/lekkasjer i forhold til avføring/luft at du må forsikre deg om hvor nærmeste toalett er? *(Kryss av ett svaralternativ)*

(a)

Aldri	☐ 0
Sjelden	☐ 1
Av og til	☐ 2
Vanligvis	☐ 3
Alltid	☐ 4

(b) Hvor mye plager dette deg?
Sett kryss ved et tall mellom 0 *(ikke i det hele tatt)* og 10 *(svært mye)*

☐ 0 ☐ 1 ☐ 2 ☐ 3 ☐ 4 ☐ 5 ☐ 6 ☐ 7 ☐ 8 ☐ 9 ☐ 10

ikke i det hele tatt **svært mye**

21 Påvirker dine uhell/lekkasjer i forhold til avføring/luft din planlegging av hverdagen? *(Kryss av ett svaralternativ)*

(a)

Aldri	☐ 0
Sjelden	☐ 1
Av og til	☐ 2
Vanligvis	☐ 3
Alltid	☐ 4

(b) Hvor mye plager dette deg?
Sett kryss ved et tall mellom 0 *(ikke i det hele tatt)* og 10 *(svært mye)*

☐ 0 ☐ 1 ☐ 2 ☐ 3 ☐ 4 ☐ 5 ☐ 6 ☐ 7 ☐ 8 ☐ 9 ☐ 10

ikke i det hele tatt **svært mye**

22 Gjør dine uhell/lekkasjer i forhold til avføring/luft at du holder deg hjemme oftere enn du ønsker? *(Kryss av ett svaralternativ)*

(a)

Aldri	☐ 0
Sjelden	☐ 1
Av og til	☐ 2
Vanligvis	☐ 3
Alltid	☐ 4

(b) Hvor mye plager dette deg?
Sett kryss ved et tall mellom 0 *(ikke i det hele tatt)* og 10 *(svært mye)*

☐ 0 ☐ 1 ☐ 2 ☐ 3 ☐ 4 ☐ 5 ☐ 6 ☐ 7 ☐ 8 ☐ 9 ☐ 10

ikke i det hele tatt **svært mye**

23 Alt i alt, hvor mye vil du si at dine uhell/lekkasjer i forhold til avføring/luft griper inn i ditt dagligliv? Sett kryss ved et tall mellom 0 *(ikke i det hele tatt)* og 10 *(svært mye)*

☐ 0 ☐ 1 ☐ 2 ☐ 3 ☐ 4 ☐ 5 ☐ 6 ☐ 7 ☐ 8 ☐ 9 ☐ 10

ikke i det hele tatt **svært mye**

Livskvalitetsskår: sum skår 19a-23

24 Bruk plassen under til å beskrive eventuelle bekymringer du har i forhold til uhell/lekkasjer av avføring/luft, hva du tror er årsaken til uhellene/lekkasjene, eller andre ting du synes vi bør vite om.

Tusen takk for at du har svart på spørsmålene.
